# The qualitative and quantitative analysis of the coupled C, N, P and Si retention in complex of water reservoirs

**DOI:** 10.1186/s40064-016-2836-7

**Published:** 2016-07-22

**Authors:** Lilianna Bartoszek, Piotr Koszelnik

**Affiliations:** Department of Environmental Engineering and Chemistry, Faculty of Civil and Environmental Engineering and Architecture, Rzeszów University of Technology, al. Powstańców Warszawy 6, 35-959 Rzeszow, Poland

**Keywords:** Water reservoirs, Mass balance, Biogenic elements

## Abstract

The Solina–Myczkowce complex of reservoirs (SMCR) accounts about 15 % of the water storage in Poland. On the base of historical (2004–2006 years) data, the mass balance of nitrogen, phosphorus, total organic carbon and dissolved silicon were calculated. Large, natural affluents were the main source of the biogenic compounds in the studied ecosystem, delivering 90 % of TOC, 87 % of TN and 81 % of TP and DSi load. Moreover, results show that SMCR is an important sink for all the analysed biogenic elements. About 15–30 % of external loads were retained in the reservoir mainly in upper Solina. Due to the intensive processes of primary production, inorganic forms of nitrogen and phosphorus were mainly retained. Internal production of organic matter lead to an amount of the organic matter deposited in the sediments greater than was anticipated on the basis of the mass balance calculations. A constant load of dissolved silicon originating only from natural sources did not contribute to supplement deficits of Si present in the body of water in the reservoirs, promoting disturbances in N:C:P:Si ratios and another growth condition for other types of algae.

## Background

During the last decades an important development of anthropogenic sources of biogenic substances in supplied natural water was observed. However that growth is not similar between specific elements. Anthropogenic sources of nitrogen and phosphorus compounds are connected with sewage and fertilizer loss, while human sources of silicon are minor. Moreover increased N and P loads may stimulate primary and secondary production thus growth of organic carbon (TOC) loads (Zeleňáková et al. 2012; Wiatkowski et al. 2015; Gajewska 2015). This effect distorts the natural water C:N:P:Si ratio causing negative changes in the biology of the ecosystems and the deterioration of water quality. That is especially evident in the case of stagnant waters. The role of lakes and reservoirs as sinks of biogenic elements along the aquatic route from land to oceans is evident (Hejzlar et al. 2009; Bouwman et al. 2013; Grizzetti et al. 2015; Wiatkowski et al. 2015). The mechanism of that retention is complex and connected with physical, hydrological and chemical processes e.g. denitrification (for N), sedimentation and adsorption and also uptake. Qualitative and quantitative analysis of coupled C, N, P and Si retention in reservoirs and knowledge of these element cycles is essential for our understanding of the ecosystem biogeochemistry (Bouwman et al. 2013). Additionally, this information is important for interpretation of greenhouse gasses (e.g. N_2_O, CO_2_ and CH_4_) emission from reservoirs causes and intensity (Gruca-Rokosz and Tomaszek 2015).

Retention of biogenic elements in water ecosystems is frequently described as a function of the morphometric and hydrologic parameters of reservoirs. The most frequently considered factor in model studies is a hydraulic retention time (HRT); also depth and area of the reservoir and loads (Behrend and Opitz 2000; Seitzinger et al. 2002; Tomaszek and Koszelnik 2003; Hejzlar et al. 2009) are utilised. In general, HRT changes proportionally, as longer stoppage of waters in slow flow zones promotes two basic mechanisms of retention, i.e. uptake by organisms and sedimentation. The behaviour of nitrogen is slightly different, as its retention may be high also in shallow and flowing basins due to conditions promoting denitrification, which in the literature is referred to as a third nitrogen retention mechanism, understood as a difference between inflow and outflow of load (Seitzinger et al. 2002; Tomaszek and Koszelnik 2003).

The purpose of the study is interpretation of how a mountain complex of reservoirs can modify natural biogeochemical fluxes of four major biogenic elements in the river waterway. The Solina–Myczkowce complex of mountain reservoirs (SMCR) is a perfect training field for this interpretation. During the 2004–2006 period reservoir chemistry, hydrology and catchment area management were studied intensely and independently by different institutions.

## Methods

### Study site

**Fig. 1:**
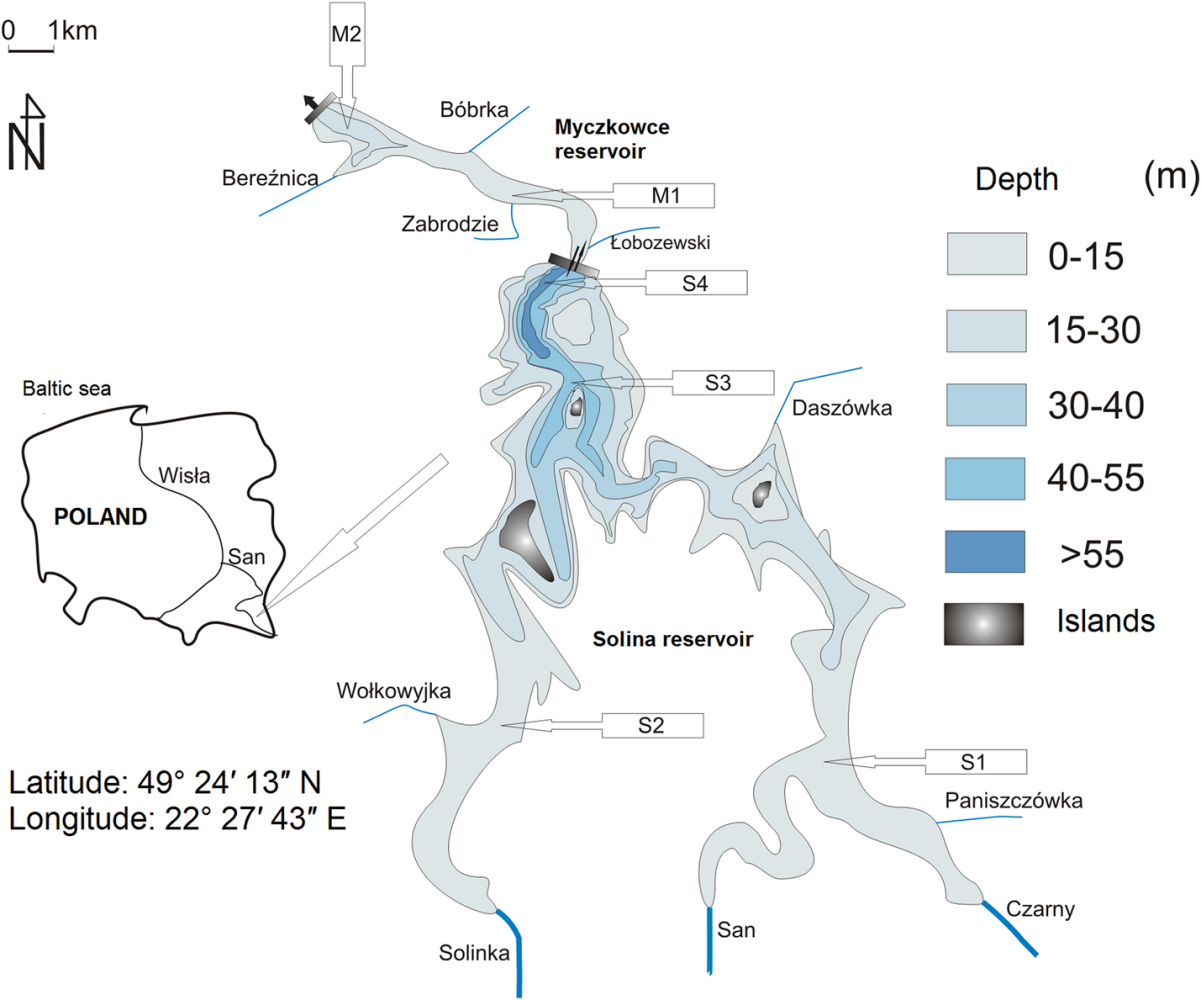
The bathymetric map of the Solina–Myczkowce complex of reservoirs. Location of sampling stations from stagnant water (M—Myczkowce, S—Solina) as well as studied affluents was shown

The SMCR is located in the upstream part of the River San (SE Poland) within the River Vistula system (Fig. 1). The upper, Solina reservoir is the biggest man-made lake in the Vistula basin and accounts for about 15 % of the total water storage capacity in Poland (volume: 502 mln m^3^, mean depth: 22 m, hydraulic retention time: 215 days, mean discharge: 35 m^3^ s^−1^). The lower Myczkowce Reservoir, as a compensatory water body (volume: 10 mln m^3^, mean depth: 5 m, hydraulic retention time: 6 days), is supplied by the hypolimnetic waters of the upper one (90 % of total supply), and by minor tributaries. The upper reservoir has three major inflows (accounting for 90 % of total water supply) and three minor ones. Reversible pumping takes place sporadically. The outflow from the complex involves bottom water from the lower reservoir flowing through a hydro-electric-power plant.

The greater part (c. 75 %) of the 1250 km^2^ catchment area is covered by forest, followed by meadows and pastures. Arable land accounts for only a small fraction of the area. The drainage basin has a low population density of about 6 inhabitants per km^2^, while about half of the households concerned are not connected to either sewerage or septic systems. A relatively steep (6 %) slope favours the leaching of soil and ground cover, especially during periods of intensive atmospheric precipitation and snow melting.

### Sampling strategy and methods

In order to assess the mass balance of N, P, DSi and TOC, water was sampled from the mouth parts of rivers and streams feeding the Solina reservoir, as well as from the outflow. Also water samples obtained from four stations on the Solina reservoir and two on the Myczkowce reservoir were subjected to analysis. Water was sampled 33 times every 1–6 weeks. Sampling dates were adjusted on the basis of the meteorological and hydrological factors. About 0.5 dm^3^ of glass-fiber-membrane-filtered water was made subject to spectrophotometric determinations for concentrations of nitrate-nitrogen (N-NO_3_^−^, salicylate method, coefficient of variation for the procedure—CVP: ±1.5 %), nitrite-nitrogen (N-NO_2_^−^, Griess reaction, CVP: ±1.7 %), ammonium-nitrogen (N-NH_4_^+^, Berthelot’s reaction, CVP: ±1.4 %), phosphate-phosphorus (P-PO_4_^3−^, Molybdate method, CVP: ±1.8 %) dissolved silicon (the Molybdate method, CVP: ±1.6 %) and chlorophyll *a* (only in stagnant water, in the methanol) using a PhotoLab S12 spectrophotometer (WTW GmBH). Moreover, Kjeldahl nitrogen (N_Kjeld_, distillation), Total Organic Carbon (TOC, Shimadzu TOC analyzer, CVP: ±0.6 %) as well as total phosphorus (TP, oxidation to phosphate), were analyzed in non-filtered samples. Total nitrogen (TN) was calculated as the sum of nitrate- and nitrite-nitrogen and N_Kjeld_. Balances of respective elements, both for the entire complex and the two reservoirs, were calculated as follows:

1 $$L_{R} + L_{DD} + L_{At} = L_{Out} + R(E)$$

where (mass time^−1^): *L*_*R*_—load inflowing from the drainage basin in affluent waters; *L*_*DD*_—load from the direct drainage basin; *L*_*At*_—load supplied from atmosphere along with precipitation; *L*_*Out*_—load removed along with run-off; R(E)—retention (elimination) of an element in the ecosystem.

Load inflowing from the drainage basin in affluent waters (*L*_*R*_) constituted a sum of loads inflowing with all the rivers and streams feeding the balanced ecosystems. Loads of elements for the particular sections were calculated as a product of concentration and water flow rate. Daily flow rate (Q) values of the analysed sections, necessary for the calculation of loads from the three main affluents (about 90 % of total supply) and run-offs from the reservoirs were obtained from Solina–Myczkowce Power Plant S.A (continuous stage measurements. Q value for smaller watercourses was calculated on the day of water sampling, using installed staff gauge readings. Concentrations between the sampling days were calculated using the statistical approach, according to Mukhopadhyay and Smith (2000). Uncertainty of calculated loads was approximated on the base of Harmel et al. (2006) as cumulative uncertainties of potential sources of error. We assumed errors during sampling (uncertainty of 15 %), chemical procedures and analyses (above mentioned CVP, ca. 2 %) and flows measurement (continuous stage measurements, uncertainty of 2 %). Therefore, C, N, P and Si loads are calculation with errors of ±19 %. To estimate the cumulative probable uncertainty of calculated retentions the root mean square error propagation method were used (Harmel et al. 2006). However, the analysis and discussion of data are based on the most probabilistic, average values due to clarity of the paper and *per analogiam* to many other authors (e.g. Mengis et al. 1997; Garnier et al. 1999; Torres et al. 2007; Hejzlar et al. 2009).

L_*DD*_ was calculated with as a sum of (1) load inflowing from point sources within the direct drainage basin (including the WWTP); (2) load inflowing from nonpoint sources within the direct drainage basin; (3) load introduced by bathers. Respective summands were calculated from the available models (Giercuszkiewicz-Bajtlik 1990; Jørgensen 2011) including data on the direct drainage basin area development, touristic burden and amount of wastewater discharged from the WWTP obtained from the Solina Municipality with its seat in Polańczyk. L_At_ was obtained from parallel study (Urbanik 2007, MSc thesis, unpublished data) utilising precipitation rate measurements carried out by the hydrological survey station in Lesko. As the reservoirs are not located in the area affected by ground waters, this source of feed was disregarded. In addition, because of the mesotrophic nature of waters, the effect of atmospheric nitrogen binding by cyanobacteria was recognised as insignificant for the balance of this element (Ferber et al. 2004; Koszelnik et al. 2007).

The sedimentation rate of TN, TOC was calculated on the basis of the phosphorus mass balance (which, in contrast to nitrogen and carbon, is not present in a gaseous state in the biogeochemical cycle), using the following formulae (Dudel and Kohl 1992):

2 $$N_sed = P_ret {-}\varDelta P (N:P)$$

3 $$C_sed = N_sed (C:N)$$

where: *N*_*sed*_ (*C*_*sed*_)—nitrogen (carbon) sedimentation rate (t year^−1^); *P*_*ret*_—phosphorus retention in the reservoir between the sampling time points (t year^−1^); N:P—ratio of total N:P concentration in benthic deposits (from Koszelnik 2009a, b); *C:N*—ratio of organic C:total N concentration in benthic deposits (from Koszelnik 2009a, b); *∆P*—change of mean phosphorus content in the water body between the sampling time days (from Koszelnik 2009a, b; t year^−1^); calculated from the following formula:

4 $$\Delta P = \frac{{\left( {P_{n + 1} - P_{n} } \right)V_{R} }}{\Delta t} \cdot \frac{365}{{10^{6} }}$$

where *P*_*n*+*1*_ and *P*_*n*_ correspond to TP concentration at nth and n + 1th day of sampling (g P m^−3^), *V*_*R*_ is a reservoir volume (m^3^), *∆t* is a time period between nth and n + 1th day of sampling (day), and 10^6^ and 365 are conversion factors to obtain the t year^−1^ unit;

## Results

**Fig. 2:**
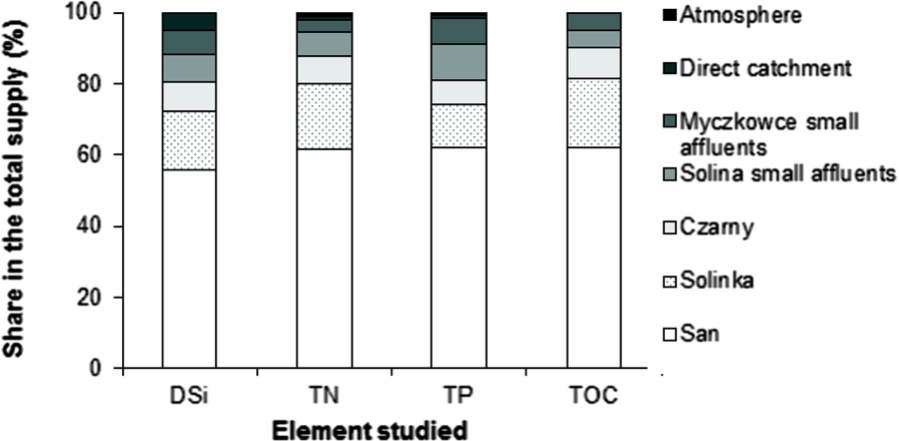
The share of different biogenic element sources in the Solina–Myczkowce complex of reservoirs supply

The results of the mass balance for the dammed reservoir complex Solina–Myczkowce are listed in Table 1. The complied data show that the inflow of TN to the reservoir from all the sources in 2004 and 2005 amounted to approx. 1700 t. In the following, last year of study, the value was ca. 50 % greater, and amounted to 2489 t. Soluble forms, in particular nitrate(V) nitrogen (ca. 60 %) were the main contributors to the load. For the other contributors loads calculated for each year of balance were less variable. Annual inflow of TP was within a range of 76–101 t; RSi 2056–2244 t; and TOC 2517–2837 t. Predominantly the loads fed the Solina reservoir. Natural affluents of the Myczkowce reservoir played a minor part in biogenic compound feed (3–7 %). Large, natural affluents (Fig. 1) were the main source of the biogenic compounds in the studied ecosystem, delivering 90 % of TOC, 87 % of TN and 81 % of TP and DSi load (Fig. 2). The share of inflows from the direct drainage basin is more evident in the case of DSi balance (5 %), whereas the load originating from atmosphere is immaterial in the annual balance.

**Table 1:** Mass balance of N, C, P and Si selected compounds for Solina and Myczkowce reservoirs

Studied compound	Year	Solina				Myczkowce				Complex of reservoirs			
		Input (t year^−1^)	Output (t year^−1^)	Retention (%)	Elimination (%)	Input (t year^−1^)	Output (t year^−1^)	Retention (%)	Elimination (%)	Input (t year^−1^)	Output (t year^−1^)	Retention (%)	Elimination (%)
Total nitrogen	2004	1702	1528	174 (10)		1536	1579		43	1711	1579	132 (8)	
	2005	1750	1313	437 (25)		1260	1323		63	1697	1323	374 (22)	
	2006	2419	2509		90	2579	2647		68	2489	2647		158
Nitrate nitrogen	2004	1021	940	81 (8)		1044	970	74 (7)		1059	970	89 (8)	
	2005	1102	892	210 (19)		819	798	21 (3)		1159	798	361 (31)	
	2006	1510	1562		52	1779	1761	18 (1)		1610	1761		151
Ammonia nitrogen	2004	136	163		27	144	158		14	137	158		21
	2005	144	176		32	149	150		1	151	150	1 (0,6)	
	2006	206	257		51	204	194	10 (5)		208	194	14 (7)	
Total phosphorus	2004	92	71	21 (23)		79	76	3 (4)		101	76	25 (25)	
	2005	72	43	29 (40)		48	51		3	76	51	25 (33)	
	2006	84	67	17 (20)		74	77		3	95	77	18 (19)	
Phosphate phosphorus	2004	42	26	16 (38)		28	29		1	46	29	17 (40)	
	2005	35	19	16 (46)		21	35		14	39	35	4 (10)	
	2006	39	22	17 (44)		25	24	1 (4)		40	24	16 (40)	
Dissolved silica	2004	1938	1603	335 (17)		1728	1748		20	2167	1748	419 (19)	
	2005	1868	1834	34 (2)		1916	1564	352 (18)		2056	1564	492 (24)	
	2006	2036	1924	112 (5)		2025	2142		117	2244	2142	102 (5)	
Total organic carbon	2004	2491	2354	137 (5)		2493	2304	189 (8)		2589	2304	285 (11)	
	2005	2813	2277	536 (19)		2402	2226	176 (7)		2837	2226	611 (22)	
	2006	2477	2141	336 (14)		2272	2087	185 (8)		2517	2087	430 (17)	

All values of Input and output are calculated with errors ±19 %

Except for TOC (R^2^ = 0.85–0.90; p < 0.001), no significant relations between hydraulic flows and concentration of total forms of the analysed elements in water were discovered. No seasonal changes in N and P concentration indicate that both N and P originate from point and non-point sources. Occurrence of these changes in the case of regional-originating Si can be related to its assimilation in river waters upstream of the reservoirs (Humborg et al. 2000).

Calculated values of loads, retention/elimination rates of N, P, Si and C are listed in Table 1. An apparent discrepancy between results from both the reservoirs and the total balance, results from inclusion of reverse pumping of water from the Myczkowce to Solina reservoir in partial balances, which influences particular balances, but has no effect on the balance for the reservoir complex. Significant masses of the balanced elements were found retained within the analysed ecosystem. This indicates that the majority of biogenic compounds feeding the reservoirs is incorporated into the trophic chain and/or accumulated in the benthic deposits (Behrend and Opitz 2000; Koszelnik et al. 2007) by various retention mechanisms. The overall reservoir balance reveals that only for nitrogen was the outflowing load higher than the inflowing load in 2006. In general, retention of biogenic compounds in the entire cascade depended on the amount of elements retained in the upper reservoir. The lower reservoir, due to a short HRT and unfavourable thermal conditions normally has neither retained nor eliminated significant amounts of biogenic compounds. Only in 2005 the silicon retention was predominantly present in the Myczkowce reservoir, with the similar relation true for N-NO_3_^−^ and TOC for the entire study period.

Except for 2006, when the SMCR was the nitrogen source for downstream waters, the nitrogen retention level was corresponding to the values determined in previous studies, carried out with varying frequency between 1970 and 2003 (10–20 %, Tomaszek and Koszelnik 2003). Approximately 60 % of the TN load supplying the reservoirs was nitrate(V) nitrogen. In 2004, from 132 t of TN retained in the reservoir complex, N-NO_3_^−^ amounted to as much as 89 t, and in 2005 the ratio was 374 to 361 t, respectively. Significantly higher load of TN in 2006 was connected rather with intensive water discharge during early spring than new nitrogen source (Urbanik 2007—unpublished data).

The determined TP loads and percentages of element retention in the reservoir complex during the balanced years were slightly less diversified than nitrogen. 19–33 % of the inflowing TP was retained in the reservoirs. Despite the fact that phosphates constitute slightly more than 50 % of the load inflowing to the reservoirs, the calculated balance reveals, that retention of the easily assimilative phosphorus form was prevailing.

Determined retention of the dissolved silicon (DSi_ret%_) in the reservoir complex varied from 5 % (2006) to 24 % (2005) of the annual external load. It was observed that in the first and the last year of the study the majority of the supplied load was accumulated in the Solina reservoir. On the contrary, in 2005 from 492 t of the retained silicon, as much as 352 t was retained in the Myczkowce reservoir. Retention of TOC was observed in both the reservoirs and ranged from 11 to 22 % of the supplied load. In a way similar to the totals of remaining elements, a major amount of carbon was retained in the studied reservoir complex in 2005.

Upon analysis of the morphometric factors on retention, a significant influence of HRT on retention of N, P and Si in waters of the entire reservoir complex was observed (Table 2). Flow rate of inflowing waters was correlated only with N_ret%_ in the Myczkowce reservoir, while the load of elements supplied to the reservoir influenced the N retention in the Myczkowce reservoir and DSi_ret%_ in the Solina reservoir, as well as in the entire complex. Much better correlations were observed upon analysis of the influence of hydraulic retention on the elements retention (W_ret_—water inflow reduced by water outflow). Increase in W_ret_ led to significant rise of N_ret%_ in both the reservoirs and the complex, DSi_ret%_ and OWO_ret%_ in the Solina reservoir and the complex and P_ret%_, but only in the balance of the entire reservoir complex (Table 1). Significant correlations between retention values of N, P and Si for the Solina reservoir and the entire complex were seen upon analysis of interrelations of retention values for all the analysed elements (Table 2). Such relations were not seen for the Myczkowce reservoir, there was no influence of TOC_ret%_ on N_ret%_, and P_ret%_ was correlated only with DSi_ret%_.

**Table 2:** Relationship between of the C, N, P, Si retention values and chosen parameters of the studied reservoirs expressed as the Pearson’s correlation coefficient with its statistical significance

	N_ret%_			P_ret%_			DSi_ret%_			TOC_ret%_		
	Solina	Myczkowce	SMCR	Solina	Myczkowce	SMCR	Solina	Myczkowce	SMCR	Solina	Myczkowce	SMCR
N_ret%_	1	1	1									
P_ret%_	0.5195^b^	–	0.6388^a^	1	1	1						
DSi_ret%_	0.4829^b^	–	0.7065^a^	0.3661^c^	–	0.6949^a^	1	1	1			
TOC_ret%_	–	–	–	–	–	–	0.3659^c^	–	0.5234^b^	1	1	1
L_Input_	–	0.4243^b^	–	–	–	–	0.3470^c^	–	0.3591^c^	–	–	–
Q	–	0.4629^b^	–	–	–	–	–	–	–	–	–	–
HRT	–	0.3871^c^	0.3345^c^	–	–	0.3292^c^	0.4579^b^	–	0.5740^a^	–	–	–
W_ret_	0.3299^c^	0.5731^a^	0.3770^c^	–	–	0.3621^c^	0.5389^a^	–	0.6111^a^	0.3387^c^	–	0.3909^c^

Abbreviations as in the text

^a^ p < 0.001; n = 36

^b^ p < 0.01; n = 36

^c^ p < 0.05; n = 36

Average annual sedimentation rate of TN, TOC in the Solina reservoir, calculated from the mass balance and levels of individual elements in sediments is provided in Table 3. Sedimentation in the Myczkowce reservoir was negligible due to the very short HRT in this reservoir (approximately 2 days). Production of sediments in this reservoir is mainly related to macrophyte production and supply of external matter. The TOC sedimentation calculated for the entire Solina reservoir amounts to 1300 t per year, and TN 110 t per year. Thickness analysis of the matter deposited from the beginning of reservoir’s existence shows that approximately twice as much of the sediments were formed in backwaters (S1, S2 stations) of the reservoir when compared to lacustrine deep areas (S3, S4 stations). This information was taken into consideration upon calculation—it was anticipated that 2/3 of the total sedimentation takes place in the said area.

**Table 3:** Sedimentation rate and content of total nitrogen, total organic carbon in the Solina reservoir

	Content in 0–2 cm thin sediment layer		Rate of sedimentation			
	Backwaters (tones)	Lacustrine (tones)	Whole Solina res. (g m^−2^day^−1^) (t year^−1^)		Backwaters (t year^−1^)	Lacustrine (t year^−1^)
TN	33	32	0.014	110	73	37
TOC	481	297	0.162	1300	870	430

## Discussion

The hydrologic balance (Urbanik 2007—unpublished data) shows that within the 3 years of study the hydraulic inflow to the reservoir was compensated by the outflow, so the calculated element retention is not the result of hydrologic factors, but biogeochemical factors. Mass balance of N, C, P and Si, conducted separately for both the reservoirs and the entire complex enabled identification of the classical biogeochemical cycle of conversion of the analysed elements. The results of the mass balance show that the SMCR retains a significant amount of biogenic elements (Table 1). The major part of elements was retained mostly in the Solina reservoir. The biogenic compounds were retained sporadically in the Myczkowce reservoir due to the hydrologic factors, i.e. feeding of a hypolimnion with N, C, P and Si-reach waters. The relationships presented in Table 2 show that retention of biogenic elements within the reservoir complex (mostly the Solina reservoir) results not only from the hydrologic or functional factors of the power station (storage of waters during the spring period and discharging during low water periods in summer and autumn), but also from inclusion of easily assimilative forms to the trophic chain and various chemical transformations. The fact that correlation between TN, TP and DSi, which are retained mostly in the easily assimilative forms (Table 1), is significant, it confirms that mechanisms of assimilation by water organisms are crucial for retention of elements in the studied reservoirs.

**Fig. 3:**
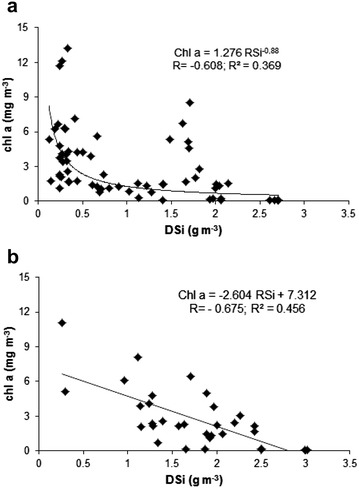
Influence of DSi depletion on phytoplankton growth (Chl *a*) in the Solina (**a**) and Myczkowce (**b**) reservoirs

**Fig. 4:**
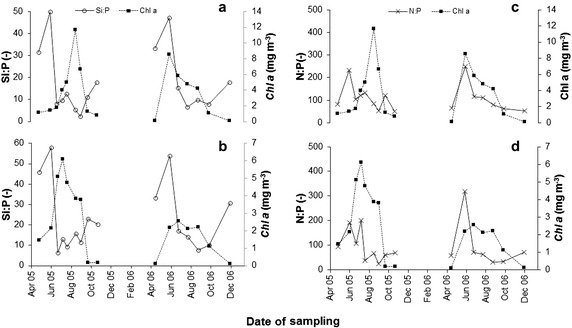
Mean chlorophyll a concentration versus N:P:Si Redfield ratios in lacustrine zone of the Solina reservoir (**a**, **c**) and Myczkowce reservoir (**b**, **d**)

The distinctive feature of the mass balance was DSi depletion from the water body in the reservoirs, mostly in the Solina reservoir (Fig. 2). Approximately 20 % of the inflowing load of DSi was retained in the studied reservoirs. Humborg et al. (2006) reports that the DSi load flowing off from 1 km^2^ of the River Vistula basin to the Baltic Sea amounts to 0.8 t per year. Average annual load of DSi feeding the SMCR amounts to 1947 t, which is equivalent to 1.5 t of flow off from 1 km^2^ of the basin. Hence, anticipating that silicon originates only from sources connected with soil erosion (Garnier et al. 1999; Humborg et al. 2002) and that the DSi load level produced in other areas of the Vistula basin is similar, ca. 50 % of dissolved silicon is retained within the area of the Vistula basin, unfavourably reducing loads feeding the Baltic sea. This decrease leads to deterioration of seawater quality due to the deficiency of DSi when compared to other biogenic compounds from anthropogenic sources. The said phenomenon leads to imbalance between diatoms and other algae (Humborg et al. 2000). A similar decrease in DSi load in dammed reservoirs was described by Garnier et al. (1999), while Humborg et al. (2006) conclude that the cascade design of dammed reservoirs on rivers increases HRT and favours retention of dissolved silicon, depleting it from downstream waters. 20 % of DSi retention is a distinctive feature of oligotrophic waters (Garnier et al. 1999), but the data available in the literature (Garnier et al. 1999; Humborg et al. 2002, 2006) also show that similar levels of DSi retention were seen in both oligo- and eutrophic ecosystems. In the studied case, depletion of dissolved silicon in the surface lake body, related in turn to DSi_ret_ affects the water quality, stimulating growth of algae. The correlations shown on Fig. 3 may confirm that increase of chlorophyll level can be related with emergence of non-diatomic (green) algae. Diatoms are seen in lake and reservoir waters mostly in spring, but also even in late winter (Humborg et al. 2000; Lehmann et al. 2004). By analogy to the condition of Lake Lugano (Lehmann et al. 2004) it can be concluded that DSi level exceeding 0.7 g m^−3^ in the epilimnion of the Solina reservoir contributes to chl *a* level related with the presence of diatoms and green algae. Below this level, in summer, a rapid increase in chl *a*, reaching even as much as 12 mg m^−3^, is observed which, in turn, may lead to occurrence of thermophilic cyanobacteria with concomitant disappearance of diatoms. Despite low water temperature in the Myczkowce reservoir, an elevated level of chl *a* was seen, but the index of >2.5 mg m^−3^ was noted only in 2005, while in 2006 it was low. In general, phytoplankton production in this reservoir is minor. However, due to the poor silicon feed, silicon shortages can occur, which lead to minor tides of algae, mostly in the warmer water area of the dam, analogous to those present in the Solina reservoir (Koszelnik 2013). A decrease in water DSi below 1 g m^−3^ was seen in 2005, but not in 2006. In addition, when silicon was almost completely depleted from the water body, a decrease in Si:N and Si:P ratios (see Fig. 4) was seen, and DSi became the limiting element. With silicon shortage present, phosphorus and nitrogen are the main substrates utilised in production of organic matter in the reservoirs. In turn, the value of N:P molar ratio (Fig. 4) significantly exceeding 16:1 proves the stoichiometric excess of nitrogen versus phosphorus.

Approximately 28 % of phosphorus supplied to the studied reservoirs is retained. The process is occurring mostly in the Solina reservoir. This result depends on various factors allowing storage of phosphorus from the water body in the benthic deposits, related in general with an affinity to specific metals, presence of aerobic conditions or with pH (Golterman 1998). Phosphorus retention is a result of sedimentation of solid particles introduced to the reservoir with affluents and assimilative forms incorporated to the biomass of phytoplankton and transferred to sediments (Hejzlar et al. 2009; Dunalska et al. 2013). A part of such retained phosphorus can be released again to the water body due to resuspension or decomposition of bonds with iron or other metals in anaerobic conditions. The benthic deposits of the Solina reservoir are rich in metals with affinity to phosphorus, mostly in iron. Retention volume of these deposits is very high (Bartoszek et al. 2009), and favourable aerobic conditions make release of phosphorus from the deposit practically absent in both the reservoirs. On that basis it can be concluded that the principal phosphorus retention mechanism in the reservoir is a direct sedimentation of phosphorus contained in suspension and intermediate sedimentation of mineral forms, after their assimilation into the trophic chain. Storage properties of the benthic deposits are large enough so that the calculated P_ret_ value could be greater, but the morphometry of the Solina reservoir (high depth, low area of an active bed) determines the identified level. Consequently, upon balancing of average phosphorus mass retained in the reservoir it was anticipated that the phosphate phosphorus retention will be equal to the amount of this element utilised by phytoplankton, and the difference between retention of P-PO_4_^3−^ and TP will be equal to the amount of element originating from external sources and accumulated in the deposit:

**Table:** 

For the complex of reservoirs (t year^−1^)	Inflow	Retention	Partial P sedimentation	Sedimentation P accumulated in biomass
	91	23	11	12

A complexity of biogeochemical nitrogen changes in the water environment affects the retention level of this element. In contrast to Si and P, nitrogen has a larger gaseous phase, and denitrification leading to change in state of aggregation affects the mass balance of this element in water ecosystems. In the studied period of 3 years, decrement of load inflowing to the reservoirs amounted only to 9 % per year. This value was affected by a significant element elimination in 2006. In 2005 N_ret%_ was equal to ca. 22 % of the introduced load. Studies on nitrogen retention, carried out between 1999 and 2003, reveal that the retention of this element was varying significantly, amounting to from 12 to 36 % of the annual load (Tomaszek and Koszelnik 2003). Previous studies show that the rate of denitrification in the Solina reservoir is stable and amounts to ca. 5 g m^−2^ year^−1^ (Koszelnik et al. 2007), which corresponds to ca. 20 % of retention and 5 % of nitrogen load. Empirical models of denitrification in conditions occurring in the Solina reservoir, contingent on presence of nitrates and temperature (Gruca-Rokosz 2005, PhD thesis, unpublished data), were utilised to analyse various nitrogen retention mechanism. Estimated denitrification rate was 4 g N m^−2^ year^−1^, and on that basis it was calculated that, on average, 70 t of N is denitrified annually. Nitrogen sedimentation calculated from the balance mass equals to 110 t year^−1^, hence the balance of retained nitrogen is as follows:

**Table:** 

For the complex of reservoirs (t year^−1^)	Inflow	Retention	Sedimentation	Denitrification	Indefinite
	1957	174	110	70	−6

 Share of denitrification process in nitrogen retention amounted to 40 % and was twice as high as that calculated for previous years (Koszelnik et al. 2007). However, load reduction is significantly lesser (similar to N_ret%_) and was only 3.6 %.

Influence of denitrification on the nitrogen mass balance depends on various factors. Seitzinger et al. (2002) describes that for North American estuaries 50 % of nitrogen is supplied from denitrification. Estuaries are bodies of water similar in many cases to dammed reservoirs; mostly due to the ratio between areas of basin and water table, retention time or biogenic compounds load. The said value can be real, but in many cases—including estuaries—significantly lower values are seen, i.e. 5–30 % (Dudel and Kohl 1992; Koszelnik et al. 2007; Povilaitis et al. 2012), but also values as high as 70 % are reported (Mengis et al. 1997). A comprehensive analysis of the available data presented in previous papers (Koszelnik et al. 2007) enables one to conclude that in water regions with high hydraulic dynamics contribution of nitrification to the mass balance is minor when compared to natural lakes, where this process can be significant.

Nitrogen sedimentation, in a way similar to phosphorus, is a result of its consumption. Thus, calculation of N_sed_ and N_cons_ contributions can be difficult. Jickells et al. (2000) states that in inland waters, retention mainly consists of storage of organic nitrogen produced within the ecosystems in the benthic deposits. In the studied case retention of assimilative forms, mostly nitrates, is equal to ca. 100 t year^−1^. Nevertheless, it should not be anticipated that the overall mass of retained NO_3_^−^ will be assimilated. Some part of it will be denitrified, as the main substrate for the said process, occurring in the anoxic layer of the benthic deposits, are nitrates(V) diffusing from water (Tomaszek and Gruca-Rokosz 2007). A surplus nitrogen seen in the above balance (−6 t) can result from utilisation of nitrogen stored in the deposits in the nitrification process, which, after various transformations, can be denitrified.

The mass balance of total organic carbon calculated for the reservoir complex shows that annually approximately 442 t of TOC is retained. The calculated TOC sedimentation is three times higher and amounts to 1300 t year^−1^. Hence, it should be recognised that a significant part of sedimentation matter is produced within the ecosystems:

**Table:** 

For the complex of reservoirs (t year^−1^)	Inflow	Retention	Sedimentation
	2206	442	1300

Lentic waters are characterised by a high carbon retention capability, as the major part of TOC supplied to lakes and reservoirs is respirated and included in the trophic chain (Garnier et al. 1999). The above is true for both deep and shallow reservoirs. Anderson and Sobek (2006) provide an example of a shallow lake in Sweden, in which the annual carbon load amounts to approximately 3 t. Calculated phytoplankton production for the lake is as high as 53 t C per year, and the macrophyte production—16 t C per year, while carbon sedimentation is three times greater than the carbon inflow to the ecosystem. Unlike the other elements, TOC retention in the Myczkowce reservoir was fairly high (7–8 % of the load), which can be explained by carbon uptake by macrophytes after its respiration. Rate of decomposition for these forms is low, and annually they release only 40 % of the retained organic carbon (Gessner 2001).

## Conclusions

Both the SMCR reservoirs are loaded with nitrogen and phosphorus in amounts significantly exceeding theoretical values considered to be allowable. Although the easily assimilative inorganic forms are dominating in the supplied mass of elements, concentration of both of the biogenic compounds and the amount of chlorophyll *a* fall within the level specific for mesotrophy. However, the inflow of biogenic elements is so significant that no distinct, seasonal variations of nitrogen and phosphorus concentrations were seen in the reservoirs. The major part of the biogenic compound load supplied to the studied reservoirs is retained therein via utilisation as substrates in the primary production process.

During the summer, dissolved silicon deficits were observed in waters in both the reservoirs. This phenomenon was present due to silicon consumption within the water body and reduced inflow from the basin. In this case silicon became a limiting element for the production of diatomic organic matter, especially in the warmer Solina reservoir. This effect was accompanied by an increase in chlorophyll *a* concentration, sporadically reaching the value specific for eutrophy, which can be related to production of other species of (non-diatomic) algae.

Retention values of particular elements, calculated from the mass balance, prove the intensity of element uptake process carried out by organisms. Approximately 20 % of inflowing total forms of N, P and Si are accumulated, mostly in the Solina reservoir. Dissolved inorganic forms are retained in more than 50 % of cases. Sedimentation of autochthonous biogenic forms is a result of inclusion of supplied elements to the trophic chain. The denitrification rate of nitrogen amounts to 20 % of retention and only to 5 % of the supplied load. TOC retention at the level of 30 % proves the allochthonous matter is accumulated in the deposit.

Sedimentation of allochthonous organic matter calculated from the mass balance is three times lower than the value estimated on the basis of the overall TOC sedimentation in the Solina reservoir. The remaining amount results from an intra-ecosystemic production stimulated by an external inflow of biogenic compounds and elements circulation within the reservoirs.
